# Efficacy of Chlorhexidine Chips as Local Drug Delivery in Nonsurgical Management of Chronic Periodontitis: A Systematic Review

**DOI:** 10.7759/cureus.73059

**Published:** 2024-11-05

**Authors:** Khaled Mashoor Hyderah, Siraj D Khan, Mahdi Mana M Alzamanan, Naif Ahmad M Alaajam, Fawaz Saad S Al Kayraan, Faisal Mohammed M Al Jally

**Affiliations:** 1 Department of Preventive Dentistry, Faculty of Dentistry, Najran University, Najran, SAU

**Keywords:** chlorhexidine chip, chronic periodontitis, local delivery, probing pocket depth, randomized clinical trials (rcts).

## Abstract

This systematic review assesses the efficacy of local chlorhexidine (CHX) chips as an adjunct to nonsurgical scaling and root planing (SRP) in treating chronic periodontitis, compared to SRP alone. A comprehensive search strategy was developed to identify relevant studies, focusing on articles published in English. Searches were conducted in MEDLINE (via PubMed), Web of Science, and periodontology journals up to December 2020, specifically looking for studies on the use of CHX chips alongside SRP for managing chronic periodontitis. This research took place from January to September 2024. The review found that sites treated with SRP plus CHX chips showed improvements in probing pocket depth, clinical attachment level, plaque index, and gingival index. The results revealed that 48.50% of the findings were not statistically significant between the two treatment groups. In contrast, 25.75% of the findings were significant for the CHX chips group compared to the SRP alone group, while 1.5% were significant for the control group. Additionally, 24.25% of the data were unavailable. Combining SRP with CHX chips results in more significant improvements in treating chronic periodontitis.

## Introduction and background

Periodontitis is an inflammation of the periodontium that damages the connective tissue that teeth attach to and extends beyond the gingiva. Periodontal diseases are generally accepted to be caused by bacteria attached to dental plaque [1]. One of the most popular treatments for periodontal diseases is scaling and root planing (SRP), against which other methods are evaluated, with adjunct treatments including local applications of chlorhexidine (CHX) and antibiotics [2].

However, in certain conditions, such as when the bacterium has penetrated the gingival tissue or is located in deep periodontal pockets, nonsurgical mechanical treatment cannot eradicate the subgingival bacteria. The outcome is bacterial recolonization, which slows down the healing process of the periodontal tissues [3]. Mechanical debridement of the tooth surface is a common treatment for periodontal disease to disturb the microbiota and create a clean, biologically acceptable root surface. However, in furcation areas and deep pockets, the effectiveness of mechanical debridement is limited. Incomplete mechanical debridement results from restricted access. Antimicrobial therapy options have therefore emerged in conjunction with mechanical debridement [4]. In particular, the local delivery of antimicrobial agents alone [5,6], and in combination with nonsurgical SRP, has been tested [7,8]. However, there are certain limitations to the effectiveness of SRP, especially in inaccessible areas. To overcome these limitations, local antimicrobial agents are often used subgingivally as adjuncts to SRP, and CHX was evaluated in randomized, blinded, and multi-center studies [9].

However, CHX solutions, whether given as a topical rinse or as an irrigant, have generally been ineffective in treating chronic periodontitis [8,10,11]. This is likely due to the inability to obtain biologically significant concentrations of the drug for sufficient periods within the confines of the periodontal pocket [12,13]. Controlled-release antibiotics administered locally offer certain benefits over those administered systemically, such as the ability to achieve high local drug concentrations in periodontal pockets and the avoidance of drug compliance issues [14]. Studies on the nonbiodegradable CHX controlled-release local delivery system indicate that when used alongside SRP, it effectively reduces bleeding on probing, improves clinical attachment level (CAL), and reduces probing pocket depth (PPD) [15,16].

This systematic review assesses the efficacy of local CHX chips as an adjunct to nonsurgical SRP in treating chronic periodontitis, compared to SRP alone.

## Review

Our review followed the Preferred Reporting Items for Systematic Reviews and Meta-Analyses (PRISMA) guidelines. We conducted both qualitative and quantitative analyses to assess the treatment outcomes.

Focused question

What is the efficacy of local CHX chips as an adjunct to nonsurgical SRP compared to the effectiveness of mechanical treatment alone in treating chronic periodontitis?

Selection criteria

We used criteria to select studies that met our eligibility requirements. This systematic review will focus on randomized controlled trial (RCT) studies evaluating the effectiveness of local CHX chips as an adjunct to SRP compared to SRP alone. It will specifically involve adults aged ≥20 years with chronic periodontitis who are nonsmokers and without systemic disease that might have affected the progression of the disease. In vitro studies will be excluded.

Search strategy

A comprehensive search strategy was developed to identify studies for this systematic review. Articles published in English will be considered. Electronic searches were conducted in PubMed, Web of Science, and journals up to December 2023. The selection strategy was based on a combination of keywords, including “chronic periodontitis”, “chlorhexidine”, “local delivery”, “probing pocket depth”, and “RCTs”.

Selection of included studies

We used citation manager software (Zotero 6.0.36, Corporation for Digital Scholarship, Vienna, Virginia, USA) to merge the available studies we found in the electronic database to remove duplicates. Two independent reviewers (KMH and SDK) then screened the titles, abstracts, and full texts of the identified articles. Potentially eligible studies were fully assessed and classified as included if they met the selection criteria.

Data extraction

Independently, four trained reviewers (MA, NA, FA, and FA) extracted relevant data into evidence tables, including author, year of publication, type of study, population characteristics, age range, sample size, study duration, and assessed periodontal parameters. The reviewers’ disagreements were resolved by discussion with KMH and SDK. From the 11 studies that made up this systematic review, data were extracted.

Results

Search and Screening

**Figure 1:**
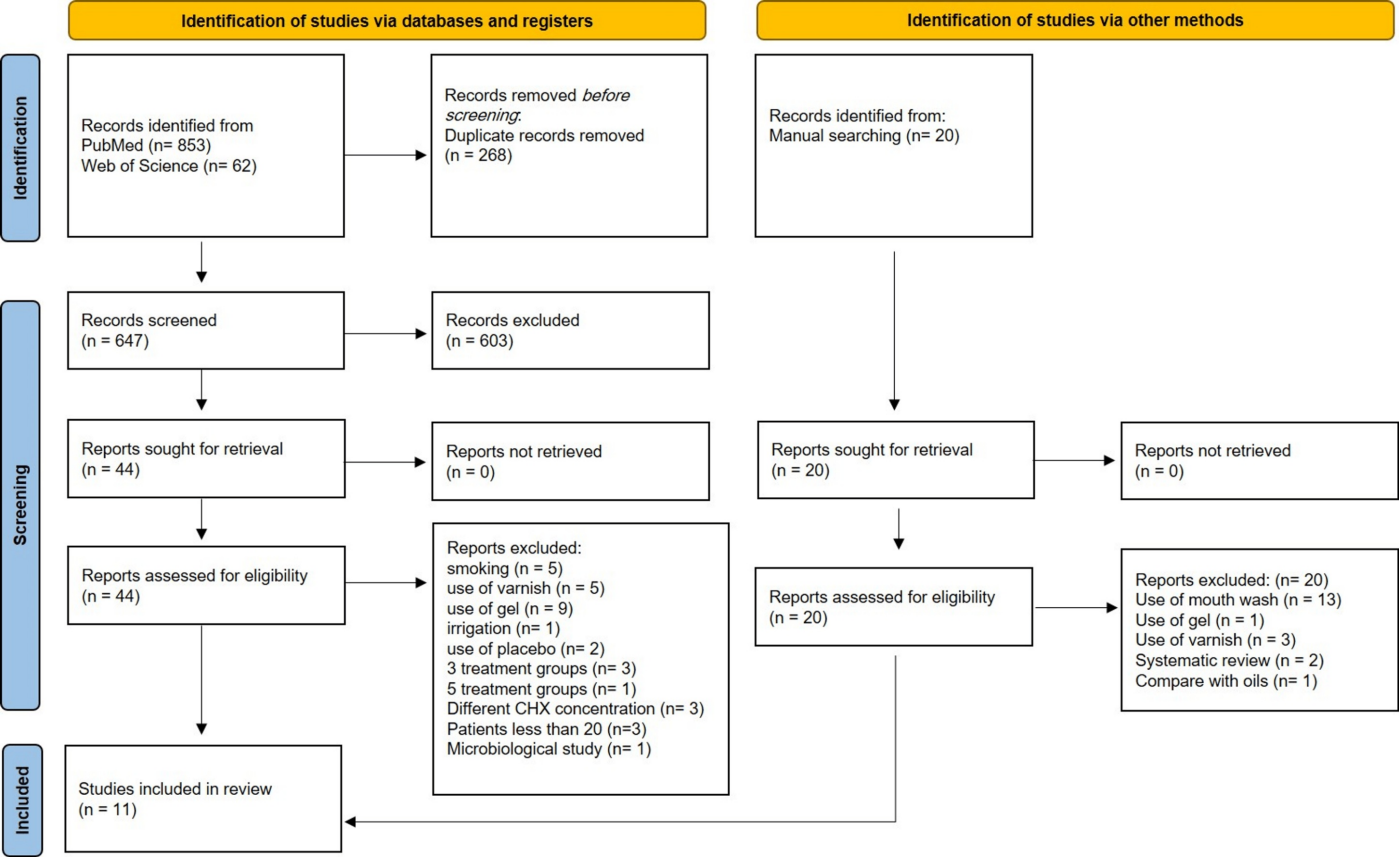
PRISMA flow diagram of the literature search and screening process PRISMA, Preferred Reporting Items for Systematic Reviews and Meta-Analyses

The search strategy yielded 935 relevant articles. After screening the titles and abstracts, 44 articles met the inclusion criteria. During the full-text assessment, 33 articles were excluded for various reasons; these reasons include smoking, CHX varnish, gel, irrigation, placebo, different CHX concentrations, and more than two treatment groups. Finally, 11 studies published between 2001 and 2015 were included [17-27] (Figure 1).

Data Analysis

Data were systematically compiled into evidence tables, allowing a comprehensive summary to identify key similarities and differences between the studies. For all of the parameters evaluated, several differences were noted between the studies, including clinical parameters, study methodologies, follow-up period assessments, sample size, and statistical analysis. These discrepancies emphasize the importance of careful consideration when drawing collective conclusions, and a meta-analysis was not possible due to heterogeneity. However, descriptive data analysis provided clinically relevant insights.

Risk of Bias Assessment of Selected Studies

All studies were randomized clinical trials. According to the guidelines of the Cochrane Collaboration, nine studies showed a high risk of bias [17,18,21-27], and two studies had an unclear risk of bias [19,20] (Table 1).

**Table 1:** Summary of risk of bias of included RCTs RCT, randomized controlled trial

Author and year of publication	Adequate sequence generation?	Allocation concealment?	Blinding?	Incomplete outcome data?	Free of selective reporting?	Free of other bias?	Overall risk
John et al. (2015) [17]	Yes	Yes	Yes	Yes	No	Unclear	High
Pattnaik et al. (2015) [18]	Yes	Yes	Yes	No	No	Unclear	High
Paolantonio et al. (2008) [19]	Yes	Yes	Yes	Unclear	Yes	Unclear	Unclear
Rodrigues et al. (2007) [20]	Yes	Yes	Yes	Yes	Unclear	Unclear	Unclear
Mızrak et al. (2006) [21]	Yes	No	No	No	No	Yes	High
Grisi et al. (2002) [22]	Unclear	Unclear	Yes	Yes	Unclear	Unclear	High
Kondreddy et al. (2012) [23]	Unclear	No	No	No	Unclear	Yes	High
Heasman et al. (2001) [24]	Unclear	Unclear	Unclear	Yes	No	Unclear	High
Kasaj et al. (2007) [25]	Yes	No	Unclear	Unclear	No	Unclear	High
Azmak et al. (2002) [26]	Yes	Unclear	Unclear	No	Yes	No	High
Grover et al. (2011) [27]	Yes	No	No	No	No	No	High

Description of Studies

Characteristics of included studies: Among the studies included in this systematic review, seven utilized a split-mouth design [17-19,23-26], while four employed a parallel design [20-22,27]. Eight studies implemented a single-blinded design [17,19-22,24-26], and two utilized a double-blind method [18,27]. One study did not provide details regarding masking [23]. Additionally, one study reported on a multicenter approach [19]. The follow-up periods varied across the studies: seven studies reported a follow-up duration of six months [19-21,23-26], one study had a follow-up of nine months [22], two studies reported a follow-up of three months [18,27], and one study had a follow-up of 11 weeks [17]. Overall, the duration of follow-up among the studies ranged from a minimum of one month to a maximum of nine months. The studies were conducted at various university institutions, with four studies taking place in India [17,18,23,27], two in Turkey [21,26], two in Brazil [20,22], and one study each in Italy, the United Kingdom, and Germany [19,24,25] (Table 2).

**Table 2:** Characteristics of included studies RCT, randomized controlled trial; U, university

Author and year of publication	Study design	Type of periodontitis	Country/setting	Masking	Informed consent	Follow-up
John et al. (2015) [17]	RCT split-mouth	Chronic periodontitis	India U	Single-blinded	Yes	3 months 0, 11 days, 11 weeks
Pattnaik et al. (2015) [18]	RCT split-mouth	Moderate to severe chronic periodontitis	India U	Double-blind	Yes	3 months 0, 1, 3 months
Paolantonio et al. (2008) [19]	RCT split-mouth multicenter study	Advanced periodontitis	Italy 4 U	Single-masked	Yes	6 months 0, 3, 6 months
Rodrigues et al. (2007) [20]	RCT parallel	Chronic periodontitis	Brazil U	Single-blinded	Yes	6 months 0, 6 weeks, 3, 6 months
Mızrak et al. (2006) [21]	RCT parallel	Chronic periodontitis	Turkey U	Single-blinded	Yes	6 months 0, 1, 3, 6 months
Grisi et al. (2002) [22]	RCT parallel	Chronic periodontitis	U Brazil	Single-blinded	Yes	9 months 3, 6, 9 months
Kondreddy et al. (2012) [23]	RCT split-mouth	Chronic periodontitis	India U	NA	Yes	6 months 0, 3, 6 months
Heasman et al. (2001) [24]	RCT split-mouth	Moderate to severe chronic periodontitis	UK U	Single-blinded	Yes	6 months 0, 1, 3, 6
Kasaj et al. (2007) [25]	RCT split-mouth	Moderate-to-severe chronic periodontitis	Germany U	Single-blinded	Yes	6 months 0, 1, 3, 6 months
Azmak et al. (2002) [26]	RCT split-mouth	Moderate to severe chronic periodontitis	Turkey U	Single-blinded	Yes	6 months 0, 1, 3, 6 months
Grover et al. (2011) [27]	RCT parallel	Mild to moderate chronic periodontitis	India U	Double-blind	Yes	3 months 0, 1, 2, 3

Population Characteristic

Patient characteristics: A total of 394 patients, with a minimum of 20 and a maximum of 116 patients per study, aged 20-70 years, were evaluated in the included studies. Two studies did not report the patient’s gender [23,26]. Four studies diagnosed patients with moderate to severe chronic periodontitis [18,24-26], while five studies identified chronic periodontitis [17,20-23], One study diagnosed mild to moderate chronic periodontitis [27], and one study identified advanced periodontitis [19]. Due to the use of systemic antibiotics during the studies, one patient was excluded from one study, and two patients were excluded from another study [22,26]. Additionally, two patients failed to attend examinations during two consecutive time frames in one study [27], and 14 patients could not be examined during at least one of the periods [20], Finally, two patients withdrew after the three-month visit for reasons unrelated to treatment [24].

Teeth and site characteristics at baseline: The studies reported 394 teeth and 948 sites with chronic periodontitis, 460 sites treated with SRP plus CHX chips, and 488 treated only with SRP. Two studies focused on treating molars [18,23], while two other studies only addressed single-rooted teeth [20,26]. Two studies involved molars, premolars, and anterior teeth [17,24]. Teeth with furcation involvement were excluded in two studies [19,25], and two studies did not specify the types of treated teeth [22,27]. One study did not report the number of sites or the types of teeth treated [21] (Table 3).

**Table 3:** Patient and teeth characteristics BANA, N-benzoyl D, L-arginine-2naphthylamide test; BI, bleeding index; BOP, bleeding on probing; CAL, clinical attachment level; F, female; GBI, gingival bleeding index; GCF, gingival crevicular fluid; GI, gingival index; GR, gingival recession; M, male; mGI, modified gingival index; PBI, papillary bleeding index; PBS, papillary bleeding score; PI, plaque index; PPD, probing pocket depth; RAL, relative attachment level; SRP, scaling and root planing

Author and year of publication	Mean age/range (years)	Patients (total and m/f)	Dropout	Teeth/site	PPD	Clinical parameters assessed	CHX chip application interval	Conclusions
John et al. (2015) [17]	41.8 ± 5.6 35 - 56	21 2/9	0	Premolar and molar SRP plus chip (20 sites) SRP alone (20 sites)	6-7 mm	PPD, CAL, GR, PI, GI, and BOP	PerioCol^TM^CG 2.5 mg, 1x at baseline	GI at three months showed statistically significant differences for the CHX chip group.
Pattnaik et al. (2015) [18]	40.9 5± 7.56 29 - 54	20 9/11	0	First molar SRP plus chip (20 sites) SRP alone (20 sites)	≥6 mm	PPD, CAL, GI, and bacterial count	PerioCol^TM^CG 2.5 mg, 1x at baseline	PPD showed statistically significant differences at 1, 3 months, and CAL at 3 months for the CHX chip group.
Paolantonio et al. (2008) [19]	NA 33-65	116 17/41	0	Anterior, premolar SRP plus chip (116 sites) SRP alone (116 sites)	≥5 mm	PPD, CAL, PI, mGI, BOP, and bacterial count	Periochip 2.5 mg, 1x at baseline	PPD and CAL showed statistically significant differences at 3 and 6 months for the CHX chip group.
Rodrigues et al. (2007) [20]	44.7 ± 11.6 30 - 70	56 24/32	14	Single-rooted teeth SRP plus chip (28 sites) SRP alone (28 sites)	5-8 mm	PPD, CAL, GR, PI, GI, and BOP	Periochip 2.5 mg, 1x at baseline	There are no significant differences between treatment groups for PPD, CAL, PI, and GI at any time point.
Mızrak et al. (2006) [21]	35 ± 8.5 20 - 55	35 5/6	0	NA	5-8 mm	PPD, CAL, GR, PI, GI, GBI, GCF, bacterial count, and biochemical analysis	Periochip 2.5 mg, 1x at baseline and 3 months	PPD showed statistically significant differences at 3, and 6 months, CAL at 6 months, and PI at 1,3, and 6 months for the CHX chip group.
Grisi et al. (2002) [22]	41.8 ± 5.6 35-56	21 2/9	1	NA SRP plus chip 41 site SRP alone 39 site	≥5 mm	PPD, RAL, PBS, GR, PI, BOP, and BANA	Perio-Chip^A^ 2.5 mg, 1x at baseline, 3, and 6 month	CAL showed statistically significant differences at 3, and 6 months for the control group, and GI at 3 months for the CHX chip group.
Kondreddy et al. (2012) [23]	NA 35-55	20	0	Posterior teeth SRP plus chip (20 sites) SRP alone (20 sites)	≥5 mm	PPD, CAL, PI, and BOP	PerioCol^TM^CG 2.5 mg, 1x at baseline	CAL and PI showed statistically significant differences at 3, and 6 months, and GI at 6 months for the CHX chip group.
Heasman et al. (2001) [24]	NA 34 - 59	26 4/9	2	Molar, premolar, anterior SRP plus chip (135 sites) SRP alone (165 sites)	At least 5 mm	PPD, CAL, PI, and BI	PerioCol^TM^ 2.5 mg, 1x at baseline	CAL and GI showed statistically significant differences at 6 months for the CHX chip group.
Kasaj et al. (2007) [25]	42.0 ± 5.6 20 - 60	20 7/13	0	Anterior and premolar SRP plus chip (40 sites) SRP alone (40 sites)	≥5 mm	PPD, CAL, GR, PI, GI, and BOP	PerioChip 2.5 mg, 1x at baseline and 3 months	PPD and CAL showed statistically significant differences at 1, 3, and 6 months, and GI at 6 months for the CHX chip group.
Azmak et al. (2002) [26]	NA 36 - 62	22	2	Single-rooted tooth SRP plus chip (20 sites) SRP alone (20 sites)	6-8 mm	PPD, CAL, PBI, and PI	Periochip 2.5 mg, 1x at baseline	There are no significant differences between treatment groups for PPD, CAL, PI, and GI at any time point.
Grover et al. (2011) [27]	38.6 35 - 54	42 1/3	2	NA SRP plus chip (20 sites) SRP alone (20 sites)	5-8 mm	PPD, CAL, BI, and radiological (bone gain)	PerioCol^TM^CG 2.5 mg, 1x at baseline	PPD and CAL showed statistically significant differences at 1, and 3 months, and GI at 3 months for the CHX chip group.

Treatment prior to the baseline: In all the studies, supragingival scaling and polishing were performed, and oral hygiene instructions were provided to participants before their baseline visit. In five studies, supragingival scaling and instructions were given two weeks before baseline [19-21,25,26], while in two studies were provided one week before baseline [17,27]. One study was conducted two to four weeks before baseline [24], and in three studies it was not reported [18,22,23].

Parameter Outcomes

PPD reductions: In a comprehensive analysis of PPD reductions across multiple follow-up periods, the findings were as follows: at the one-month follow-up, data from six studies indicated that three studies showed significant improvements in the SRP plus CHX chip group compared to SRP alone [18,25,27], while the other three studies did not report significant differences [21,24,26]. At the three-month follow-up, 11 studies were analyzed, revealing that five studies reported significant differences in PPD reductions for the SRP plus CHX chip group [18,19,21,25,27]. At the six-month follow-up, eight studies were reviewed, with three studies finding significant differences favoring the SRP plus CHX chip group [19,21,25]. However, five studies did not show significant differences [20,22-24,26]. Additionally, data from one study indicated that the differences in PPD reduction between the treatment groups were not statistically significant at nine months [22]. In three studies, all pockets that remained ≥5 mm in depth received subgingival SRP, while the test sites received SRP and new CHX chips [21,22,25], as observed after three months and in one study after six months [22].

CAL gains: In a comprehensive analysis of CAL gain across multiple follow-up periods, the findings were as follows: at the one-month follow-up, data from six studies indicated that two found statistically significant differences favoring the SRP plus CHX chip group over SRP alone [25,27], while four studies did not show significant differences [18,21,24,26]. At the three-month follow-up, an analysis of 11 studies revealed five studies with significant differences favoring the SRP plus CHX chip group [18,19,23,25,27], one study showing significant differences for the control group over the test group [22], and five studies with no significant difference [17,20,21,24,26]. At the six-month follow-up, eight studies were analyzed, with five reporting significant differences for the SRP plus CHX chip [19,21,23-25], one study indicating significant differences for the control group [22], and two studies showing no significant differences [20,26]. Finally, at the nine-month follow-up, data from one study indicated no statistically significant differences in CAL gains between the treatment groups [22].

Gingival inflammation improvement: To evaluate the improvement in gingival inflammation among different groups at one, three, and six-month follow-up, data from 11 studies were analyzed [17-27]. Two studies found no statistically significant differences between the treatment groups at any time point [21,26]. Additionally, two other studies reported no significance at three and six months [19,20], while one study showed no significance at one and three months [18]. Conversely, six studies reported statistically significant differences in gingival inflammation for the SRP plus CHX chip group compared to the SRP alone group [17,22-25,27]. Among these six studies, two found significance at three months [17,27], while three studies reported significant differences at six months [23-25]. Additionally, one study used the papillary bleeding score (PBS), which showed significant results at three months but not at six months [22].

Plaque index (PI) improvement: To evaluate the improvement in PI among different groups at one-, three-, and six-month follow-ups, data from 11 studies were analyzed [17-27]. Nine studies reported no statistically significant differences between treatment groups [17-20,22,24-27]. Among these nine studies, three found no statistical significance between treatment groups at any time point [24-26], three reported no significance between treatment groups at three and six months [19,20,22], two found no significance between treatment groups at one and three months [18,27], and one found no differences at three months [17]. Regarding the SRP plus CHX chips group, studies reported significant differences between treatment groups at one, three, and six months [21] and three and six months [23].

Adverse events: Local adverse events were observed, with the most common findings in CHX-treated sites being gingival discomfort at three sites and gingival swelling at one site [25]. In another study, gingival abscesses were identified at three sites [22]. Furthermore, five subjects from the CHX chip group reported experiencing gingival pain and tenderness [27].

Discussion

This systematic review evaluates the efficacy of CHX chips in treating periodontal pockets in patients with chronic periodontitis when used alongside SRP, noting benefits such as improved CAL and reduced PPD [9,28]. However, some studies indicate limited additional benefits, potentially due to serum proteins reducing their antimicrobial effectiveness [22]. Moreover, the ability of CHX to bind to salivary bacteria minimizes bacterial repopulation and inhibits biofilm maturation, which enhances its antimicrobial action and overall treatment efficacy. POB, PPD, and CAL are essential for diagnosing periodontal disease and assessing treatment outcomes [29]. Periochip, which contains approximately 2.5 mg of CHX gluconate in a biodegradable matrix of type 1 collagen, effectively inhibits over 99% of microorganisms in periodontal pockets for more than a week, exceeding the minimum inhibitory concentration [30].

**Figure 2:**
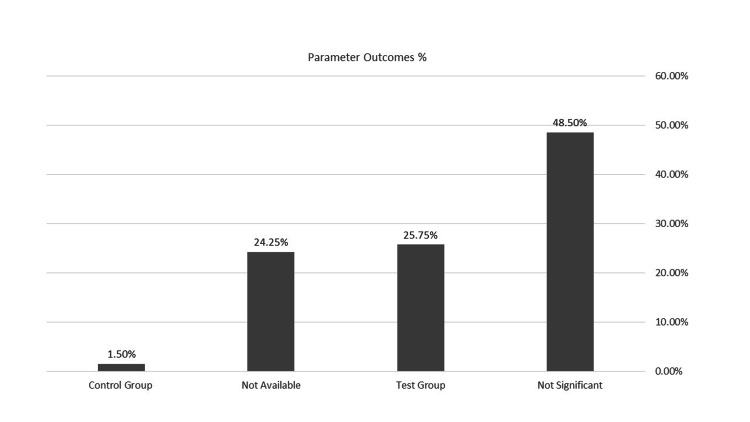
Significance level of the two treatment groups

RCTs from 2001 to 2015 were included in this analysis, and PPD, CAL, PI, and gingival index (GI) were the main outcomes assessed in the studies included in this review. The review found that the CHX chips are superior after one, three, and six months of follow-up (Table 4, Figure 2).

**Table 4:** Summary of studies outcomes at all follow-up intervals for two treatment groups * p < 0.05 ** p < 0.001 CAL, clinical attachment level; GI, gingival index; H sig., highly significant; PI, plaque index; PPD, probing pocket depth; sig., significant

Author and year of publication	PPD reduction at different periods			CAL gain at different periods			PI at different periods			GI at different periods		
	At one month	At three months	At six months	At one month	At three months	At six months	At one month	At three months	At six months	At one month	At three months	At six months
John et al. (2015) [17]	NA	Not sig.	NA	NA	Not sig.	NA	NA	Not sig.	NA	NA	H sig.	NA
Pattnaik et al. (2015) [18]	Sig.	Sig.	NA	Not sig.	Sig.	NA	Not sig.	Not sig.	NA	Not sig.	Not sig.	NA
Paolantonio et al. (2008) [19]	NA	Sig.	Sig.	NA	Sig.	Sig.	NA	Not sig.	Not sig.	NA	Not sig.	Not sig.
Rodrigues et al. (2007) [20]	NA	Not sig.	Not sig.	NA	Not sig.	Not sig.	NA	Not sig.	Not sig.	NA	Not sig.	Not sig.
Mızrak et al. (2006) [21]	Not sig.	Sig.	Sig.	Not sig.	Not sig.	Sig.	Sig.	Sig.	Sig.	Not sig.	Not sig.	Not sig.
Grisi et al. (2002) [22]	NA	Not sig.	Not sig.	NA	Sig.	Sig.	NA	Not sig.	Not sig.	NA	Sig.	Not sig.
Kondreddy et al. (2012) [23]	NA	Not sig.	Not sig.	NA	Sig.	Sig.	NA	Sig.	H sig.	NA	Not sig.	Sig.
Heasman et al. (2001) [24]	Not sig.	Not sig.	Not sig.	Not sig.	Not sig.	Sig.	Not sig.	Not sig.	Not sig.	Not sig.	Not sig.	Sig.
Kasaj et al. (2007) [25]	Sig.	Sig.	Sig.	Sig.	Sig.	Sig.	Not sig.	Not sig.	Not sig.	Not sig.	Not sig.	Sig.
Azmak et al. (2002) [26]	Not sig.	Not sig.	Not sig.	Not sig.	Not sig.	Not sig.	Not sig.	Not sig.	Not sig.	Not sig.	Not sig.	Not sig.
Grover et al. (2011) [27]	Sig.	Sig.	NA	Sig.	Sig.	NA	Not sig.	Not sig.	NA	Not sig.	Sig.	NA

During the first month of the follow-up period, the PPD change was 1.45 ± 0.59 mm [18], 1.3 ± 0.6 mm [25], and 0.47 ± 0.61 mm [27], with a statistical significance for the SRP plus CHX chip group. Additionally, the percentage of sites showing a reduction of ≥2 mm was significantly higher in the SRP plus CHX chip group compared to the SRP alone group (p < 0.05) [25]. The CAL gain was reported as 1.2 ± 0.7 mm [25] and 0.36 ± 0.76 mm [27], with a statistically significant difference observed for the SRP plus CHX chip group compared to SRP alone. The greater reduction in PPD and CAL gain in the SRP plus CHX chip group may be due to the baseline pocket depth, pocket management, and the additive effect of the CHX chip released gradually over time [18].

In the three-month follow-up period, the reduction in PPD for SRP plus CHX chip was reported as 2.36 ± 0.84 mm [18], 1.8 ± 0.8 mm [25], and 1.26 ± 1.19 mm [27], with significant difference for the SRP plus CHX chip compared to SRP alone group. One study reported a reduction from 6.94 ± 0.74 mm to 4 mm with significant results for the SRP plus CHX chip [21]. Additionally, another study found statistically significant results for the SRP plus CHX group (p < 0.01) [19]. The CAL gain for SRP plus CHX chips was reported as follows: 1.6 ± 1.0 mm [25], 1.15 ± 1.30 mm [27], 1.8 ± 0.6 mm [23], and 2.29 ± 0.50 mm [18], with statistical significance for SRP plus CHX chips. One study reported a significant CAL gain with (p < 0.01) for the SRP plus CHX chip [19]. Additionally, another study found a CAL gain of 1.4 ± 0.3 mm for SRP alone, which was statistically significant when compared to the SRP plus CHX chip group [22]. The observed improvement can be attributed to the antimicrobial effect of CHX chips [15]. Patients with deeper baseline pocket depth experienced a greater CAL gain and a greater PPD reduction following SRP [31].

During the six-month follow-up period, the PPD reduction for the SRP plus CHX chip was reported as 3.82 mm [21] and 2.2 ± 0.8 mm [25], both showing significance for the SRP plus CHX chip group. One study reported a significant PPD reduction with p< 0.01 for the SRP plus CHX chip [19]. The CAL gain at six months was measured at 2.82 mm, 3.2 ± 0.9 mm, 0.43 ± 0.15 mm, and 1.9 ± 1.1 mm for the SRP plus CHX chip group compared to the SRP alone group [21,23-25], respectively. In another study, CAL improvement at six months was significantly higher in the control group, reaching 1.4 ± 0.3 mm, compared to 0.4 ± 0.5 mm in the test group [22]. Additionally, for both SRP plus CHX chip and SRP alone, the CAL gain for 1 mm was 57.8% and 28.4% [19], respectively. Differences in study design, population characteristics, and the re-instrumentation of pockets remaining ≥5 mm may explain these findings, along with multiple applications of CHX chips.

Additionally, studies reported that the percentage of sites exhibiting a PPD reduction of ≥2 mm was significantly higher in the SRP plus CHX chip group than in the SRP alone group at one, three, and six months (p < 0.05) [25] and three and six months (p < 0.01) [19]. One study did not find statistically significant differences between the two treatment groups (p > 0.05) [20]. Site characteristics and disease severity can explain these findings. The review identified chronic periodontitis across multiple studies, including moderate-to-severe, mild-to-moderate, and advanced periodontitis.

The GI was assessed to evaluate overall oral inflammation status. In the three-month follow-up period, the results showed statistically significant improvement for the SRP plus CHX chip group, with p-values of <0.003, <0.05 for PBS, and <0.007 [17,22,27], respectively. After six months, the improvements were still statistically significant for the SRP plus CHX chips group, with p-values <0.004, <0.05, and <0.05 [23-25], respectively. This improvement can be explained by the patient’s adherence to oral hygiene instructions.

The PI was used to assess overall oral hygiene status, revealing significant differences in scores between the SRP plus CHX chip group and the SRP alone group [21,23]. The difference was statistically significant for the SRP plus CHX chip group at one, three, and six months, with a p-value of <0.05 [21]. In another study, the SRP plus CHX chip group showed significance at three and six months, with p-values of <0.001 and <0.0001 [23], respectively. This improvement can be explained by CHX’s ability to inhibit bacterial adhesion to teeth by disrupting the formation of biofilms, which prevents bacterial aggregation [32]. Interestingly, two studies in this review revealed nonsignificant results at every time point for PPD, CAL, PI, and GI [20,26]. Study design, population characteristics, and pocket depth can explain these findings.

Additionally, three studies detailed adverse events in sites treated by CHX chips [22,25,27]. This can be explained by improper placement technique for the CHX chip, insufficient preparation of surrounding tissues, and inter-examiner variability [33]. In this review, four studies performed intra-examiner and inter-examiner calibrations to minimize variability among examiners [19,20,24,25]. Variations in session length and the effectiveness of SRP may account for inconsistent treatment results. It is recognized that the thoroughness of SRP has an impact on the healing process of the pockets [34].

Other limitations include insufficient details about the duration of instrumentation, examiner calibration, and multiple applications of CHX chips. In this review, two studies received subgingival retreatment and application of CHX chips for sites ≥5 mm after three months [21,25]. Additionally, one study conducted this procedure after three and six months [22]. In a study by Kasaj et al. [25], the sites that exhibited pocket reduction ≥2 mm were 39%, 69%, and 71% for SRP plus CHX chip, and 5%, 15%, and 13% for SRP alone after one, three, and six months, respectively. As a result, examiner reliability, site characteristics, and study design may impact the variations observed between these studies. Furthermore, there are differences in treating single-rooted compared to multi-rooted teeth. These present challenges, including access to the root surface, the complexity of the root surface, the furcation area, and the consuming process of SRP.

The CHX chip biodegrades within seven to 10 days post-implantation, which may explain the observed improvement when combined with SRP during the initial stages of healing [35]. When discussing the outcomes of CHX chips as an adjunct to nonsurgical therapy, it is crucial to distinguish between the effects of different delivery methods. CHX mouthwashes have demonstrated promising clinical benefits for managing periodontal disease, particularly in reducing plaque and gingival inflammation [36]. CHX irrigation, varnish, and gel provide a short-term antibacterial effect, with a substantivity of approximately 12 hours [37].

The review notes that the included studies have incomplete information about allocation concealment, missing data, and selective reporting. The absence of these essential methodological details raises concerns about possible bias and emphasizes the need for standardized protocols to ensure reliability in the clinical or research field.

Finally, the results revealed that 48.50% of the findings were not statistically significant between the two treatment groups. In contrast, 25.75% of the findings were significant for the CHX chips group compared to the SRP alone group, while 1.5% were significant for the control group. Additionally, 24.25% of the data were unavailable.

## Conclusions

The use of CHX chips has been shown to improve plaque and gingival indices, reduce PPD, and enhance CAL, particularly in pockets deeper than 5 mm. However, further evaluation through standardized RCTs is needed to assess microbiological outcomes and establish the effectiveness of CHX chips in reducing periodontal pathogens, while ensuring studies are standardized, inclusive of diverse populations, controlled for confounding variables, include long-term follow-up, and utilize comprehensive outcome measures that incorporate clinical, microbiological, and patient-reported data.
